# Incidence of Aflatoxins and Ochratoxin A in Wheat and Corn from Albania

**DOI:** 10.3390/toxins15090567

**Published:** 2023-09-12

**Authors:** Dritan Topi, Janja Babič, Breda Jakovac-Strajn, Gabrijela Tavčar-Kalcher

**Affiliations:** 1 Veterinary Faculty, Institute of Food Safety, Feed and Environment, University of Ljubljana, Gerbičeva 60, 1000 Ljubljana, Slovenia; janja.babic@vf.uni-lj.si (J.B.); breda.jakovacstrajn@vf.uni-lj.si (B.J.-S.); gabrijela.tavcar-kalcher@vf.uni-lj.si (G.T.-K.); 2 Department of Chemistry, Faculty of Natural Sciences, University of Tirana, Boulevard Zogu 1, 25/1, 1000 Tirana, Albania

**Keywords:** aflatoxins, ochratoxin A, liquid chromatography–tandem mass spectrometry, corn, wheat, Albania

## Abstract

In this study, aflatoxins (AFs) and ochratoxin A (OTA) were analyzed in grains, specifically wheat and corn, from Albania. To summarize, 71 wheat and 45 corn samples from different growing areas were collected. The multi-toxin analytical procedure involved sample extraction and liquid chromatography–tandem mass spectrometry (LC–MS/MS). The incidence of AF was 18% in the analyzed wheat and 71% in the corn samples. The concentration of AFs was much higher in the corn samples than in the wheat samples. The maximum permitted levels for aflatoxin B1 (AFB1) and total AFs were not exceeded in the wheat samples, while they were exceeded in 36% of the corn samples. In the wheat samples, the AFB1 concentration varied between 0.2 and 0.4 µg kg^−1^. However, the highest concentrations in the corn samples were 2057, 2944, and 3550 µg kg^−1^. OTA was present in only three corn samples and one wheat sample. However, all contaminated samples exceeded the maximum permitted levels. This report reveals the presence of AFs and OTA in grain commodities, specifically wheat and corn, grown in Albania.

## 1. Introduction

Mycotoxins are secondary metabolites of low molecular weight produced by filamentous fungi, or more specifically, molds [1,2,3,4,5,6,7,8,9,10,11]. They contaminate cereals and other foods such as nuts, spices, fruits, and their by-products [8,12,13]. Contamination of cereal crops with toxigenic fungi and, hence, mycotoxins can occur in the field and during harvesting, handling, storage, and processing [1,4,6,8,9,10,11,14,15,16]. Exposure to mycotoxins in food is associated with adverse effects on humans and animals, which can be both acute and chronic [7,8,9,11,16,17,18,19,20,21]. They are considered one of the most important diet risks, which is why the content in food is regulated in the European Union [22].

Aflatoxins (AFs) are chemically classified as polyketide-derived difuranocoumarins, produced mainly by two *Aspergillus* species. *A. flavus*, the most important producer, is ubiquitous, favoring the aerial parts of plants and producing B-type aflatoxins, while *A. parasiticus* is adapted to the soil environment with more limited distribution, capable of further expanding oxidative rings to produce G-type aflatoxins [4,23,24]. Contamination of cereals with AFs occurs mainly during storage [6,7], but can also occur in the field, before and during harvesting [4,14,15,17,25].

There are four main types of AFs: Aflatoxin B1 (AFB1), aflatoxin B2 (AFB2), aflatoxin G1 (AFG1), and aflatoxin G2 (AFG2). AFB1 is considered the most toxic aflatoxin and the most potent carcinogen, classified as Group 1 (“carcinogenic to humans”) by the International Agency of Research on Cancer [26]. It is estimated that aflatoxin exposure may be responsible for 5–28% of all cases of hepatocellular carcinoma worldwide [27]. Chronic exposure to AFs in food commodities manifests immunosuppressive, genotoxic, mutagenic, teratogenic, hepatotoxic, and hepatocarcinogenic effects [4,6,7,8,17,21,24,25,28]. High levels of exposure are manifested as acute aflatoxicosis [4,6,7,8,17,21,24,25,28].

Ochratoxin A (OTA) is a natural contaminant in many foodstuffs, including cocoa beans, coffee beans, cassava flour, cereals, fish, peanuts, dried fruits, wine, and meat products such as salami and ham. It is produced by fungi of the genera *Aspergillus* and *Penicillium*. [4,29,30]. OTA possesses nephrotoxic, hepatotoxic, neurotoxic, teratogenic, and immunotoxic effects [30].

The presence of AFs and OTA in foodstuffs is regulated by Commission Regulation (EU) No. 2023/915 [22]. The maximum permitted levels of AFB1 and the sum of AFB1, AFB2, AFG1, and AFG2 in corn and rice intended for consumers or use as an ingredient in food are 5.0 and 10.0 µg kg^−1^, respectively. In other cereals, they are 2.0 and 4.0 µg kg^−1^, respectively. The maximum permitted level of OTA in unprocessed cereal grains is 5 µg kg^−1^.

Favorable growth regions for aflatoxigenic fungi belong to tropical and subtropical regions [5,28,31]. Due to climate change, AF contamination of cereal crops has been documented in southern Europe in recent decades [16,32,33,34,35,36,37,38,39]. Further reports on contamination with Afs, as well as with OTA, in cereals and cereal products from European countries have been published by Binder et al. [40], Serrano et al. [41], Bryła et al. [42], Jakovac–Strajn et al. [43], Alkadri et al. [44], Kirinčič et al. [45], EFSA [24], Pleadin et al. [46,47], Gagiu et al. [38], and Marin et al. [4]. There are several surveys on the occurrence of AFs from other parts of the world reported by Gruber–Dorninger et al. [48], Rodrigues et al. [49], Aasa et al. [50], Ayeni et al. [51], Abdallah et al. [52], Dong et al., [53], Jiang et al. [54], and Sun et al. [55].

The analytical determination of mycotoxins In food and feed commodities is carried out by a number of quantitative and semi-quantitative techniques, enzyme-linked immunosorbent assay (ELISA) [32,33,34,38,46,47], gas chromatography–mass spectrometry [7,18], and liquid chromatography coupled with different detectors, including ultraviolet [32,40,45], fluorescence [32,33,40,52,56], and mass spectrometry [35,39,41,42,44,46,54]. Liquid chromatography with fluorescence detection (HPLC–FLD) is often employed for quantitatively identifying individual mycotoxins. Their simultaneous determination is possible using a technique that combines liquid chromatography and mass spectrometry [7,18,57].

This study aimed to obtain data on the occurrence of aFs and OTA in cereals from Albania. Sample collection was carried out from different regions of the country, considering production capacity and geography. The countrywide annual output of corn, wheat, barley, and rye is 380,000, 275,000, 7500, and 3000 tons, respectively, according to data from the FAO (Food and Agriculture Organization) [58]. Accordingly, mainly wheat and corn samples were taken, but also a limited number of samples of barley and rye. The analytical procedure involved a sample extraction and LC–MS/MS. This study is the first-ever report on the presence of Afs and OTA in plant commodities from Albania. The data will expand the information on the presence of mycotoxins in different cereals from southern Europe, a region of growing interest in relation to the impact of climate change.

## 2. Results and Discussion

### 2.1. Occurrence of aFs and OTA in Grains

The grain samples containing one or more individual AF or OTA at levels above the limit of quantification (LOQ) were considered positive. Overall, 40% of the grain samples tested were contaminated. However, there were large differences in contamination between corn and wheat and between harvesting years. The contamination rate for corn was 71%, while it was only 20% for wheat. In 2014, 90% of the corn samples were contaminated, while in 2015, only 29% were. The contamination rate for wheat was 40% in 2014, but no incidence was revealed in 2015. The incidence of positive samples, mean, median, and minimum and maximum concentrations of positive samples are shown in Table 1 and Table 2.

As shown in Table 1 and Table 2, the AF levels were much higher in the corn compared to the wheat samples. While the AFB1 concentration in the wheat samples ranged between 0.2 and 0.4 µg kg^−1^, the concentrations in corn were as high as 2944 and 3550 µg kg^−1^ (all in 2014). The maximum permitted levels for AFB1 and total aFs [22] were not exceeded in the wheat samples, but they were 4 in 16 corn samples (36%). Three of them were from the 2015 harvesting year, and the other 13 samples were from 2014. Thus, more than 40% of the corn samples in 2014 did not comply with the legal requirements [22], which can pose a significant health risk for consumers. Therefore, regular and systematic control of the presence of aFs in grain commodities and actions for their reduction are crucial.

Altogether, 34% of the contaminated corn samples contained only one AF (either AFB1 or AFG2), and 38% of the contaminated corn samples contained all four aFs. Meanwhile, 13% contained two and 16% contained three aFs. AFB1 and AFG2 were present in 81% of the contaminated samples, while AFB2 and AFG1 were present in 47% of the contaminated samples. All contaminated wheat samples contained only one AF (either AFG1 or AFG2). AFG2 was present in 31% and AFG1 in 6% of the wheat samples from 2014.

In total, only three corn samples (7%) and one wheat sample (1.5%) were contaminated with OTA. However, the concentrations were high. They exceeded the maximum permitted level in all contaminated samples. The differences between the years were smaller than in the case of aFs. In 2014, the incidence of OTA in corn was 6% and in 2015 it was 7%. The maximum concentrations were 333 and 488 µg kg^−1^ in 2014 and 2015, respectively. OTA was not detected in the barley and rye samples, while in one sample of rye, AFB2 was present (0.61 µg kg^−1^).

The data on the incidence of aFs and OTA in corn commodities according to the regions are presented in Table 3. The incidence is comparable in all five regions (71–100%). Additionally, the mean concentrations were comparable in all regions. However, there were significant differences in the maximum concentrations of AFB1 and the sum of aFs among the regions.

Samples from two regions, Fieri and Lushnja, located in the western plain in the Adriatic Sea, had higher maximum levels than samples from Kruja, Elbasan, and especially the eastern region of Korça. Concerning the maximum concentrations, the order of incidence according to the areas was Lushnja > Fieri > Kruja > Elbasan > Korça. Except for Korça, the other regions are located in the western plain, characterized by a Mediterranean climate of a hot summer and a humid winter, while the eastern areas of the country are characterized by a temperate continental climate. The highest AFB1 levels were found in a sample from Lushnja (3550 µg kg^−1^) and a corn sample from the Fieri region (1232 µg kg^−1^), which are the main producing regions of corn and dairy [59].

### 2.2. Comparison with the Incidence of aFs from Other Countries

The incidence of AFB1 and aFs in corn samples in our study (58% and 71%, respectively) is comparable to the results from Serbia [33,35,56] given in Table 4, while in other studies, a lower incidence was reported [4,38,39,43,45,47,48,49,52,53,54]. In Croatia, Slovenia, Romania, the EU, Egypt, China, the Middle East, and African countries, it was 0–45.4% [4,38,39,43,45,47,48,49,52,53,54]. In a global survey on the occurrence of mycotoxins in feed performed in 100 countries, an AFB1 incidence of 24% was reported [48], while Lee and Ryu [60] reported a 55% incidence for aFs based on global occurrence data reported from 2006 to 2016. The determined concentrations were much higher than in all other countries, with the exception of the study from Croatia [47] and the worldwide occurrence [60], where comparable maximum concentrations (2072 and 1642 µg kg^−1^, respectively) were reported. However, as mentioned above, there was also a big difference between the 2014 and 2015 occurrences in Albania, where 90% of the corn samples were contaminated in 2014 and only 29% in 2015. The maximum concentrations were 4822 and 126 µg kg^−1^, respectively.

The incidence of AFB1 and aFs in the wheat samples in our study (3% and 18%, respectively) is comparable to the results from other countries, where an incidence of 0–19% was reported (Table 5), except in Romania, where the incidence was 45.4%. The maximum level of AFB1 and aFs in the wheat samples from Albania (0.4 µg kg^−1^) was much lower than in other studies, where maximum AFB1 concentrations from 5.41 µg kg^−1^ in a sample from Croatia [47] to 109 µg kg^−1^ in a sample from EU [4] and 161 µg kg^−1^ in a sample from a study on global occurrence of mycotoxins [48] were reported.

The low rate of OTA-positive corn and wheat samples is comparable to the incidence in other surveys, where it was <10% [38,39,43,45,48,49,52,54]. However, Kos et al. [35] reported an incidence of 25% and 18% in corn from Serbia in 2012 and 2015, respectively, while Marin et al. [4] reported 54% of cereal samples to be positive in the EU [4], and Lee and Ryu [60] reported a global incidence of 29% between 2006 and 2016. Nevertheless, the concentrations determined in our study are much higher than those reported in all other studies.

The occurrence of mycotoxins in cereals produced in Albania, especially aflatoxin contamination, is associated with other food commodities, such as dairy products, showing mycotoxin contamination [61]. The survey of mycotoxin contamination in the country is more and more relevant due to climate change and the country’s accession to the EU.

## 3. Conclusions

In this study, the presence of AF and OTA was determined in 125 samples from two seasons, providing the first insight into their occurrence in Albanian grains by contributing to our knowledge of the mycotoxin contamination problem in southern Europe.

The incidence of four aflatoxins in the corn samples was higher than in wheat. When compared to publications of the same period from this region, it is comparable to the incidence reported in Serbia but higher than that reported in other countries. Additionally, the concentrations were significantly higher than elsewhere. The incidence of AFB1 in wheat was similar to the incidence in the region, but its concentrations were lower.

The incidence of OTA in corn and wheat was rather low, comparable to the incidence reported in other countries, except for in a few studies, where a higher incidence was reported.

However, the main concern is the high rate of results exceeding the maximum permitted levels, which can pose a significant health risk for consumers. As mentioned, regular and systematic control of the presence of aFs in grain commodities and actions for their reduction are important. The significant difference between the data from two harvesting years, 2014 and 2015, indicates that further surveys based on harvesting years need to be conducted to adequately characterize the occurrence of aFs in cereals in Albania.

## 4. Materials and Methods

### 4.1. Sample Collection

Wheat and corn grains were sampled during harvesting seasons from five main agriculture regions: Fieri, Lushnja, Korça, Elbasan, and Kruja. Grain sampling was conducted in June–July for two harvesting years, 2014 and 2015. Corn sampling was accomplished in October for the two harvesting years. The samples were taken from the field site from small farms and warehouses. At the time of sampling or during grain growth, the weather conditions were not recorded. The Commission Regulation (EC) No. 401/2006 [62] was followed in terms of the sampling procedure to ensure representative samples. During the two harvesting seasons, 2014 and 2015, 71 wheat samples and 45 corn samples were collected. As part of this study, 35 wheat and 31 corn samples were collected in 2014, and 36 wheat and 14 corn samples were collected in 2015. In 2015, consent to collect corn samples was obtained from only 14 farms. In addition, seven barley samples and two rye samples were collected in the Korça and Fieri regions (in 2014, five barley and two rye samples; in 2015, two barley samples).

### 4.2. Standards and Chemicals

A mixed aflatoxin standard solution in acetonitrile (AFB1, AFB2, AFG1, and AFG2) was purchased from Romer Labs (Tulln, Austria). Stock standard consisting of 2 μg mL^−1^ (AFB1 and AFG1) and 0.5 μg mL^−1^ (AFB2 and AFG2) and mixed working standard solutions were stored in amber glass vials at −20 °C. Acetonitrile, acetic acid, and methanol of HPLC grade from Sigma-Aldrich (Steinheim, Germany) and ammonium acetate of p.a. grade by Merck (Darmstadt, Germany) were purchased. The Milli-Q system (Millipore, Bedford, MA, USA) was employed for deionized water production.

### 4.3. Sample Preparation

For the simultaneous determination of the four aFs, a procedure described in detail by Topi et al. [63] was employed. The procedure consisted of mycotoxin extraction from the ground cereal samples and LC–MS/MS, according to Rasmussen et al. [64], Lattanzio et al. [65], and Schenzel et al. [66]. The grains were ground to a particle size of 1 mm using a Retsch ZM 100 laboratory mill (Haan, Germany). A 10 g sample size was shaken with 100 mL of a mixture of acetonitrile and deionized water (84 + 16) for 1 h using an IKA HS 501 digital linear shaker (IKA Labortechnik, Staufen, Germany). An aliquot (4 mL) of the filtered extract was evaporated to dryness using the Syncore Polyvap system (Buchi, Flawil, Switzerland). When the mycotoxin concentration was above the calibration range, the filtered extracts were diluted for further work. The dry residue was reconstituted in a 0.5 mL mixture of deionized H_2_O and MeOH (20 + 80). Finally, 10 μL of the solution was injected into the UPLC–MS/MS “Acquity UHPLC Class System” connected to a quadrupole mass spectrometer (Xevo TQ MS) equipped with an electrospray ionization interface (ESI) and MassLynx software for data acquisition and processing (Waters, Milford, MA, USA). The vials were stored at 10 °C in the autosampler. For matrix-matched calibration, an appropriate amount of standard solution was added to a 4 mL aliquot of the filtered extract and prepared along with the sample.

### 4.4. LC–MS/MS Operation

Chromatographic separation was performed on a Zorbax Eclipse Plus C18 Rapid Resolution HD column (2.1 × 100 mm, 1.8 μm; Agilent). Aflatoxin separation was performed using mixture A (deionized water) and B (methanol) in a 60:40 ratio isocratic condition. For OTA analysis, an elution described elsewhere [63] was used. The mobile phase flow rate was fixed at 0.3 mL min^−1^, and the column temperature at 40 °C. MS/MS analysis was performed in MRM (multiple reaction monitoring) mode. The specific MS/MS parameters (retention time, ionization mode, and monitored transitions) associated with specific mycotoxins are shown in Table 6. The LOD and LOQ for the single aFs were 0.6 and 2.0 µg kg^−1^, respectively, while for OTA they were 1.5 and 5.0 µg kg^−1^, respectively.

## Figures and Tables

**Table 1:** Occurrence of aflatoxins and OTA in corn from the 2014 and 2015 harvesting seasons.

	AFB1	AFB2	AFG1	AFG2	Sum	OTA
2014						
Analyzed samples	31	31	31	31	31	31
Positive samples	23	15	15	25	28	2
Incidence (%)	74	48	48	81	90	6.0
Mean value (μg kg^−1^)	464	93.9	158	16.4	531	260
Median value (μg kg^−1^)	21.1	8.5	5.4	1.3	6.6	260
Minimum value (μg kg^−1^)	0.3	0.2	0.2	0.2	0.2	187
Maximum value (μg kg^−1^)	3550	539	978	144	4822	333
2015						
Analyzed samples	14	14	14	14	14	14
Positive samples	3	0	0	1	4	1
Incidence (%)	21	0.0	0.0	7.1	29	7.1
Mean value (μg kg^−1^)	55.7	-	-	0.2	41.8	488
Median value (μg kg^−1^)	31.7	-	-	0.2	20.6	488
Minimum value (μg kg^−1^)	9.4	-	-	0.2	0.2	488
Maximum value (μg kg^−1^)	126	-	-	-	126	488
2014–2015						
Analyzed samples	45	45	45	45	45	45
Positive samples	26	15	15	26	32	3
Incidence (%)	58	33	33	58	71	7.0
Mean value (μg kg^−1^)	417	93.9	158	15.8	469	336
Median value (μg kg^−1^)	22.2	8.5	5.4	1.3	8.5	333
Minimum value (μg kg^−1^)	0.3	0.2	0.2	0.2	0.2	187
Maximum value (μg kg^−1^)	3550	539	978	144	4822	488

**Table 2:** Aflatoxins and ochratoxin A occurrence in wheat from the 2014 and 2015 harvesting seasons.

	AFB1	AFB2	AFG1	AFG2	Sum	OTA
2014						
Analyzed samples	35	35	35	35	35	35
Positive samples	2	0	0	11	13	1
Incidence (%)	6	0	0	31	37	3
Mean value (μg kg^−1^)	0.3	-	-	0.3	0.3	611
Median value (μg kg^−1^)	0.3	-	-	0.2	0.2	611
Minimum value (μg kg^−1^)	0.2	-	-	0.2	0.2	611
Maximum value (μg kg^−1^)	0.4	-	-	0.3	0.4	611
2015						
Analyzed samples	36	36	36	36	36	36
Positive samples	0	0	0	0	0	0
Incidence (%)	-	-	-	-	-	-
Mean value (μg kg^−1^)	-	-	-	-	-	-
Median value (μg kg^−1^)	-	-	-	-	-	-
Minimum value (μg kg^−1^)	-	-	-	-	-	-
Maximum value (μg kg^−1^)	-	-	-	-	-	-
2014–2015						
Analyzed samples	71	71	71	71	71	71
Positive samples	2	0	0	11	13	1
Incidence (%)	3	0	0	15	18	1.5
Mean value (μg kg^−1^)	0.3	-	-	0.3	0.3	611
Median value (μg kg^−1^)	0.3	-	-	0.2	0.2	611
Minimum value (μg kg^−1^)	0.2	-	-	0.2	0.2	611
Maximum value (μg kg^−1^)	0.4	-	-	0.3	0.4	611

**Table 3:** Occurrence of aflatoxins and ochratoxin A in corn according to region from the harvesting year 2014.

	AFB1	AFB2	AFG1	AFG2	Sum	OTA
Fieri						
Analyzed samples	4	4	4	4	4	4
Positive samples	4	3	4	4	4	0
Incidence (%)	100	75	100	100	100	0.0
Mean value (μg kg^−1^)	315	39.6	5.08	2.50	352	-
Median value (μg kg^−1^)	12.7	1.91	2.92	2.43	18.7	-
Minimum value (μg kg^−1^)	2.82	0.20	0.22	0.82	5.65	-
Maximum value (μg kg^−1^)	1232	117	14.3	4.34	1367	-
Lushnja						
Analyzed samples	7	7	7	7	7	7
Positive samples	7	6	7	7	7	1
Incidence (%)	100	85.7	100	100	100	14
Mean value (μg kg^−1^)	1235	173	332	54.7	1795	187
Median value (μg kg^−1^)	36.2	155	7.22	3.08	46.7	187
Minimum value (μg kg^−1^)	5.46	1.79	2.39	0.2	1.0	187
Maximum value (μg kg^−1^)	3550	539	978	144	4822	187
Kruja						
Analyzed samples	7	7	7	7	7	7
Positive samples	7	5	3	6	7	1
Incidence (%)	100	71	43	86	100	14
Mean value (μg kg^−1^)	93.7	14.2	8.98	1.60	109	333
Median value (μg kg^−1^)	10.6	10.0	0.32	1.01	12.0	333
Minimum value (μg kg^−1^)	0.32	0.21	0.16	0.18	0.32	333
Maximum value (μg kg^−1^)	344	47.5	26.5	3.47	393	333
Elbasan						
Analyzed samples	6	6	6	6	6	6
Positive samples	2	1	0	5	5	0
Incidence (%)	33	17	0.0	83	83	0.0
Mean value (μg kg^−1^)	48.6	8.54	-	1.03	22.2	-
Median value (μg kg^−1^)	48.6	8.54	-	1.17	1.58	-
Minimum value (μg kg^−1^)	1.42	8.54	-	0.25	0.25	-
Maximum value (μg kg^−1^)	95.8	8.54	-	1.58	106	-
Korça						
Analyzed samples	7	7	7	7	7	7
Positive samples	3	0	1	3	5	0
Incidence (%)	43	0.0	14	43	71	0.0
Mean value (μg kg^−1^)	1.29	-	0.24	0.80	1.30	-
Median value (μg kg^−1^)	1.39	-	0.24	0.77	1.39	-
Minimum value (μg kg^−1^)	0.85	-	0.24	0.56	0.56	-
Maximum value (μg kg^−1^)	1.62	-	0.24	1.06	1.86	-

**Table 4:** Occurrence of aflatoxins and ochratoxin A in corn samples from different studies.

Country	Sampling Year	Analytical Method	LOD/LOQ (μg kg^−1^)	Mycotoxin	No. of Samples	Positive Sample Rate (%)	Mean (μg kg^−1^)	Median (μg kg^−1^)	Max (μg kg^−1^)	Reference
Albania	2014–2015	LC–MS/MS	0.6/2.0	AFB1	45	58	417 ^a^	22.2 ^a^	3550	This study
				aFs	45	71	469 ^a^	8.5 ^a^	263	
				OTA	45	7.0	336 ^a^	333 ^a^	488	
Serbia	2012	LC–MS/MS	0.33/1.0	AFB1	40	60	-	-	70.3	[33]
		ELISA	1.40/5.0							
Serbia	2015	HPLC–FLD	0.4/1.3	AFB1	180	57.2	11.4 ± 14.5	-	88.8	[56]
			-	aFs	180	57.2	12.7 ± 17.3	-	91.4	
Serbia	2012	LC–MS/MS	0.25/-	AFB1	51	94	44 ± 49	26	205	[35]
	2013				51	33	8 ± 11	5	48	
	2014				51	0	-	-	-	
	2015				51	90	8 ± 9	4	41	
	2012		0.4/-	OTA	51	25	53 ± 108	6	318	
	2013				51	2	-	-	-	
	2014				51	0	-	-	-	
	2015				51	18	6 ± 8	5	27	
Croatia	2013	ELISA	1.0/1.7	AFB1	972	31.4	38.46	-	2072	[47]
Croatia	2016	LC–MS/MS	0.3/1.0	AFB1	61	0	-	-	-	[39]
	2017				23	8.7	5.5	-	9.7	
	2016		0.3/1.0	OTA	61	0	-	-	-	
	2017				23	0	-	-	-	
Slovenia	2007–2008	HPLC–FLD	0.2/0.6	AFB1	58	0	-	-	-	[43]
			10/30	OTA	58	1.7	30	30	30	
Slovenia	2008–2012	LC–MS/MS	-/0.2	AFB1	69 ^b^	0	-	-	-	[45]
			-/0.8	aFs	69 ^b^	0	-	-	-	
			-/1	OTA	69 ^b^	0	-	-	-	
Romania	2012–2015	ELISA	-	aFs	97 ^c^	45.4	3.85 ± 14.80	<1.75	82.94	[38]
			-	OTA	97 ^c^	6.8	2.70 ± 0.43	<2.50	6.72	
EU	2000–2006	-	0.1–0.2/-	AFB1	943	14	0.26	0.12	8	[4]
			0.2–0.4/-	aFs	943	14	0.41	0.24	9	
		-	0.01–0.5/-	OTA	5180 ^c^	54	0.29	-	33.3	
Egypt	2014–2015	LC–MS/MS	0.72/2.4	AFB1	79	16	-	4.81	197.5	[52]
			2.8/9.4	OTA	79	3	-	<LOQ	11	
Middle East and	2009	HPLC–FLD	0.3/0.8	aFs	63	35	28	32 ^a^	343	[49]
African countries			0.2/30.5	OTA	1	0	-	-	-	
Shandong, China	2014–2015	UPLC–Q-TOF-MS	0.05/0.1	AFB1	520	18.08	7.62	-	573.13	[53]
Shandong, China	2016	LC–MS/MS	0.01/0.03	AFB1	90 ^b^	32.2	0.22	-	8.04	[54]
				OTA	90 ^b^	0	-	-	-	
Global	2008–2017	HPLC	0.2–2.7/-	AFB1	15,889	24	-	4	6105	[48]
		LC–MS/MS	0.2–5/-							
		ELISA	1–3/-							
		HPLC	0.06–2/-	OTA	6388	5	-	3 ^a^	889	
		LC–MS/MS	0.2–100/-							
		ELISA	0.2–2/-							
Global		-	-	aFs		55 ^c^			1642	[60]
		-	-	OTA		29 ^c^			1164	

^a^ Only positive samples. ^b^ Corn and corn-based processed foods. ^c^ Cereals, processed cereals, and cereal-based food.

**Table 5:** Occurrence of aflatoxins and ochratoxin A in wheat samples from different studies.

Country	Sampling Year	Analytical Method	LOD/LOQ (μg kg^−1^)	Mycotoxin	No. of Samples	Positive Sample Rate (%)	Mean (μg kg^−1^)	Median (μg kg^−1^)	Max (μg kg^−1^)	Reference
Albania	2014–2015	LC–MS/MS	0.6/2.0	AFB1	71	3	0.3	0.3	0.4	This study
				aFs	71	18	0.3	0.2	0.4	
				OTA	71	1.0	611 ^a^	611 ^a^	611	
Serbia	2012	LC–MS/MS	0.33/1.0	AFB1	30	0	-	-	-	[33]
		ELISA	1.40/5.0							
Croatia	2013	ELISA	1.0/1.7	AFB1	201	7.5	1.65	-	5.41	[47]
Croatia	2016	LC–MS/MS	0.3/1.0	AFB1	57	0	-	-	-	[39]
	2017				47	2.1	16.2	-	16.2	
	2016		0.3/1.0	OTA	57	0	-	-	-	
	2017				47	4	153.7	-	614	
Slovenia	2007–2008	HPLC–FLD	0.2/0.6	AFB1	20	5	0.2	0.2	0.2	[43]
			10/30	OTA	20	0	-	-	-	
Slovenia	2008–2012	LC–MS/MS	-/0.2	AFB1	80 ^b^	0	-	-	-	[45]
			-/0.8	aFs	80 ^b^	0	-	-	-	
			-/1	OTA	80 ^b^	2.5	3.9 ± 2.8	-	5.8	
Romania	2012–2015	ELISA	-	aFs	97 ^c^	45.4	3.85 ± 14.80	<1.75	82.94	[38]
			-	OTA	97 ^c^	6.8	2.70 ± 0.43	<2.50	6.72	
Italy	2009–2010	LC–MS/MS	0.2/1	AFB1	46	0	-	-	-	[44]
			0.4/1	OTA	46	0	-	-	-	
Mediterranean area	2009–2010	LC–MS/MS	-/0.25	AFB1	65	15	-	-	66.7	[41]
Poland	2014	UHPLC–HRMS	-/5	aFs	99	0	-	-	-	[42]
			-/4	OTA	99	0	-	-	-	
EU	2000–2006	-	0.1–0.2/-	AFB1	3010 ^d^	7	0.35	0.20	109	[4]
			0.2–0.4/-	aFs	3010 ^d^	7	0.51	0.40	117	
			0.01–0.5/-	OTA	5180 ^d^	54	0.29	-	33.3	
The Middle East and	2009	HPLC–FLD	0.3/0.8	aFs	32 ^e^	19	1	2 ^a^	7	[49]
African countries			0.2/30.5	OTA	1	0	-	-	-	
Global	2008–2017	HPLC	0.2–2.7/-	AFB1	2210	10	-	1	161	[48]
		LC–MS/MS	0.2–5/-							
		ELISA	1–3/-							
		HPLC	0.06–2/-	OTA	1973	9	-	3	364	
		LC–MS/MS	0.2–100/-							
		ELISA	0.2–2/-							

^a^ Positive samples. ^b^ Wheat and wheat-processed foods. ^c^ Cereal, processed cereal, and cereal-based food. ^d^ Cereals other than corn. ^e^ Wheat/wheat bran.

**Table 6:** Ionization mode, retention times, and monitored aflatoxin and ochratoxin A transitions.

Mycotoxin	Mode of Ionization	Retention Time (min)	Ion Precursor (*m*/*z*)	Quantifier Ion (*m*/*z*)	Qualifier Ion (*m*/*z*)
AFB1	ESI+	5.25	313.2	213.2	241.1
AFB2	ESI+	4.75	215.2	243.2	259.1
AFG1	ESI+	2.92	329.1	214.8	199.9
AFG2	ESI+	5.33	331.1	189.0	285.1
OTA	ESI+	11.10	404.2	221.0	239.0

## Data Availability

The data presented in this study are available on request from the corresponding author.
